# Systematic Tracing of Susceptible Animals to SARS-CoV-2 by a Bioinformatics Framework

**DOI:** 10.3389/fmicb.2022.781770

**Published:** 2022-03-04

**Authors:** Hailiang Sun, Ailan Wang, Lixia Wang, Bing Wang, Geng Tian, Jialiang Yang, Ming Liao

**Affiliations:** ^1^College of Veterinary Medicine, South China Agricultural University, Guangzhou, China; ^2^Geneis Co., Ltd., Beijing, China; ^3^School of Electrical and Information Engineering, Anhui University of Technology, Maanshan, China; ^4^Academician Workstation, Changsha Medical University, Changsha, China; ^5^Institute of Animal Health, Guangdong Academy of Agricultural Sciences, Guangzhou, China

**Keywords:** SARS-CoV-2 Variants, ACE2, molecular docking, spike protein, Omicron, evolutionary origin

## Abstract

Since the outbreak of SARS-CoV-2 in 2019, the Chinese horseshoe bats were considered as a potential original host of SARS-CoV-2. In addition, cats, tigers, lions, mints, and ferrets were naturally or experimentally infected with SARS-CoV-2. For the surveillance and control of this highly infectious disease, it is critical to trace susceptible animals and predict the consequence of potential mutations at the binding region of viral spike protein and host ACE2 protein. This study proposed a novel bioinformatics framework to systematically trace susceptible animals to SARS-CoV-2 and predict the binding affinity between susceptible animals’ mutated/un-mutated ACE2 receptors. As a result, we identified a few animals posing a potential risk of infection with SARS-CoV-2 using the docking analysis of ACE2 protein and viral spike protein. The binding affinity of some of these species is weaker than that of humans but more potent than that of Chinese horseshoe bats. We also found that a few point mutations in human ACE2 protein or viral spike protein could significantly enhance their binding affinity, posing an enormous potential threat to public health. The ancestors of the Omicron may evolve rapidly through the accumulation of mutations in infecting the host and jumped into human beings. These findings indicate that if the epidemic expands, there may be a human-animal-human transmission route, which will increase the difficulty of disease prevention and control.

## Introduction

In December 2019, Coronavirus disease 2019 (COVID-19) was first reported in Wuhan, China. The etiological agent of COVID-19 has been confirmed as a novel coronavirus, named severe acute respiratory syndrome coronavirus 2 (SARS-CoV-2) (Gorbalenya et al., 2020). COVID-19 rapidly spread and caused a global pandemic. By 22 September 2021, SARS-CoV-2 resulted in 229,543,672 human infections with 4,708,355 fatalities^1^. The virus and its variants continue to circulate globally, posing a serious threat to public health.

To date, seven coronaviruses are known to infect humans, of which HCoV-229E and HCoV-NL63 are alpha coronaviruses, while HCoV-OC43, HCoV-HKU1, SARS-CoV-1, Middle East respiratory syndrome coronavirus (MERS-CoV), and SARS-CoV-2 belong to beta coronaviruses. SARS-CoV-2 shares 80% identity with SARS-CoV-1 at the nucleic acid level. SARS-CoV-2 shares 96% nucleic acid similarity with two bats β-coronaviruses, indicating that SARS-CoV-2 may be derived from bat coronavirus (Zhou et al., 2020). Previous studies have suggested that bats are one of the major natural hosts for coronaviruses such as SARS-CoV-1 and MERS-CoV and are associated with several coronaviruses causing severe human diseases. SARS-CoV-1 was confirmed to derive from bat-origin coronaviruses (Li et al., 2005; Shi and Hu, 2008), and *paguma larvata* acted as an intermediate host during the transmission of SARS-CoV-1 to humans (Hu et al., 2017). Similarly, MERS-CoV was confirmed to derive from camel-origin coronaviruses, and dromedary camels play an important role in spreading and transmitting viruses (Alagaili et al., 2014; Meyer et al., 2014; Muller et al., 2014; Sabir et al., 2016). Thus, it is critical to reveal susceptible animals, which will favor the origin tracing, prevention, and control of SARS-CoV-2.

Besides human beings, SARS-CoV-2 can infect other mammalians, such as ferrets, cats, dogs, tigers, lions, and mink. A total of 14.7% (15/102) sera collected from cats in Wuhan from January to March 2020 were confirmed positive to SARS-CoV-2 (Zhang et al., 2020). SARS-CoV-2 can effectively replicate in cats and transmit to uninfected ones through the respiratory droplets. However, other companion pets (dogs) are low susceptible to SARS-CoV-2. SARS-CoV-2 can effectively replicate in the upper respiratory tract of ferrets and does not cause severe symptoms or fatality (Shi et al., 2020). Tigers and lions in Bronx zoo of Wildlife Conservation Society with coughing symptoms were confirmed positive to the RNA of SARS-CoV-2, and these indicated that SARS-CoV-2 could infect tigers and lions (McAloose et al., 2020). In addition, minks were confirmed to be positive for SARS-CoV-2 and died of acute interstitial pneumonia in four mink farms in Netherland during 19–20 April 2020, suggesting that SARS-CoV-2 can infect mink and cause mortality (Molenaar et al., 2020). However, the spectrum of susceptible animals to SARS-CoV-2 is still unclear.

Susceptible animals to SARS-CoV-2 can be screened through animal experiments or computational simulation methods. Experimentally, the susceptible animals were evaluated through inoculation of SARS-CoV-2 virus in biosafety level 3 lab (BSL-3) (Barry Hassan et al., 2020; Rockx et al., 2020; Schlottau et al., 2020; Shi et al., 2020), which is expensive and labor-intensive. Thus, it is urgently needed to develop computational tools for prioritizing susceptible animals of SARS-CoV-2.

As we know, receptors are the biological basis for viruses to attach to and enter host cells. SARS-CoV-2 shares a high identity with SARS-CoV-1 in the receptor-binding domain of spike protein, and their receptors are all angiotensin-converting enzyme 2 (ACE2) (Xu et al., 2020). Thus, it is feasible to identify susceptible animals of SARS-CoV-2 by computational methods utilizing the sequence and structure alignment of ACE2 proteins across animal species and the molecular docking between ACE2 protein and the spike protein of SARS-CoV-2. The sequence or structure alignment-based methods compared the sequence or structure of ACE2 protein across the different hosts. The hosts with similar ACE2 receptors (to humans or other confirmed infected species) will have a high chance of being infected by SARS-CoV-2 (Hayashi et al., 2020; Qiu et al., 2020). Protein docking-based methods directly predict the interaction between host receptor protein and the spike protein of SARS-CoV-2. The protein docking algorithms are divided into rigid and flexible ones (Sable and Jois, 2015; Lensink et al., 2016; Xu et al., 2018; Wen et al., 2019). Rigid docking methods like ZDOCK (Pierce et al., 2014) and MEGADOCK (Ohue et al., 2014) are usually simple and less time-consuming. However, their prediction reliability is also poor. On the contrary, the flexible docking methods can provide accurate prediction at the cost of high computational resources. Commonly used flexible docking algorithms include Rosetta (Wang et al., 2007), AutoDock (Morris et al., 2009), and HADDOCK (van Zundert et al., 2016). The protein docking method can not only be applied in the research of potential host search, but also in other research fields of SARS-CoV-2, such as predicting antiviral drug candidates (Dai et al., 2020), identifying SARS-CoV-2 inhibitors (Das et al., 2020; Ton and Gentile, 2020), and manifesting the molecular mechanism of SARS-CoV-2 invasion (He et al., 2020).

This study presented a novel computational framework for systematically tracing susceptible animals to SARS-CoV-2. The binding affinities of different species to the spike protein of SARS-CoV-2 were first calculated. Then, the relationship between the evolution of human ACE2 protein and the binding affinity of SARS-CoV-2 spike protein was analyzed. Finally, the influences of a few mutations at receptor binding sites of SARS-CoV-2 on its binding affinity to ACE2 proteins across different species were simulated.

## Results

### A Bioinformatics Framework for Systematically Tracing Susceptible Animals to SARS-CoV-2

**FIGURE 1:**
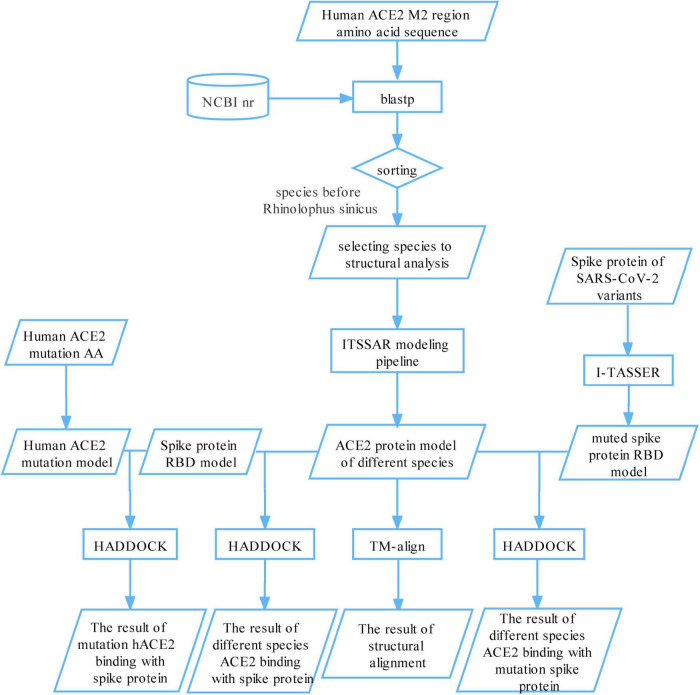
A bioinformatics framework for systematically tracing susceptible animals to SARS-CoV-2. First, we aligned the amino acids sequence of the M2 region of hACE2 protein against the NCBI nr database and selected 31 species for structural modeling and binding their ACE2 protein with SARS-CoV-2 spike protein. The binding scores of ferret, cat, and tiger were setting as a threshold to find susceptible animals to SARS-CoV-2. Second, we analyzed the binding sites of a few representative species to explain how the spike proteins interact with their ACE2 proteins. Finally, we simulated some mutations on human ACE2 protein and viral spike protein and calculated the binding affinity between mutated/un-mutated ACE2 proteins and mutated/un-mutated viral spike proteins.

We proposed a novel computational framework to trace susceptible animals to SARS-CoV-2 (Figure 1). First, we aligned the amino acids sequence of the M2 region of hACE2 protein against the NCBI non-redundant protein sequences (nr) database. Based on predefined filter rules, we identified 31 species to model the structure of their ACE2 proteins and calculated the binding affinities between their ACE2 proteins and spike protein for five SARS-CoV-2 variants, respectively. It was reported that three species, ferret, cat, and tiger, were experimentally or naturally infected with SARS-CoV-2 (McAloose et al., 2020; Shi et al., 2020; Zhang et al., 2020). Thus, we used the docking scores of the three species as the threshold values to determine susceptible animals to SARS-CoV-2. In addition, we analyzed the binding sites of a few representative species to explain how spike proteins interact with their ACE2 proteins. Finally, we simulated some mutations on human ACE2 protein and constructed the mutational human ACE2 protein model. The binding affinity prediction was performed on the mutated proteins.

### The Amino Acid Sequence of the Angiotensin-Converting Enzyme 2 M2 Region of Different Species Is similar, but That of the Angiotensin-Converting Enzyme 2 Gene Is Quite Different

Yan et al. (2020) reported that SARS-CoV-2 spike protein direct bonds to an M2 region of hACE2 protein. Thus, we extracted the amino acid sequence of the M2 region of hACE2 and aligned it with the NCBI non-redundant protein sequences (nr) database. There are 407 species with alignment identity greater than or equal to 70% (Supplementary Data Sheet 1), which all belonged to the Chordata phylum and contained 326 genera.

**FIGURE 2:**
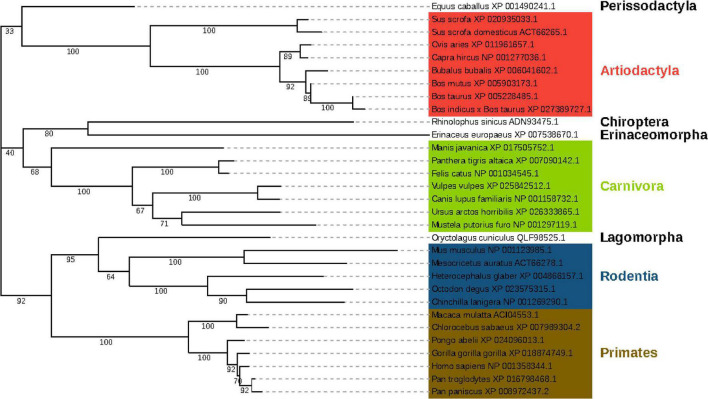
Phylogenetic tree of 31 species based on their ACE2 protein sequences. The branch length represents the number of substitutions per site, with the positions containing gaps and missing data being eliminated. The tree was clustered into 4 main orders, namely *Primates*, *Rodentia*, *Artiodactyla*, and *Carnivora*, respectively. *Primates* were marked in the red frame; *Rodentia* was in the blue frame; *Artiodactyla* was in the green frame, and *Carnivora* was in the purple frame.

We downloaded the ACE2 protein sequences of the 407 species from NCBI (Supplementary Data Sheet 2) and aligned the amino acids sequences of the M2 region of their ACE2 proteins. By setting tiger, cat, domestic ferret as thresholds and a few other criteria (see section “Materials and Methods”), we selected 30 species plus human (overall 31 species) for subsequent analysis (Supplementary Data Sheet 3). A phylogenetic tree was constructed based on the ACE2 proteins of these species using MEGA7 (Figure 2). The phylogenetic tree showed that these species clustered four main orders: Primates, Rodentia, Artiodactyla, and Carnivora. The amino acid sequence of *Primate* ACE2 protein is the closest to that of humans. The suspected host bat (*Rhinolophus sinicus*) and pangolin (*Manis javanica*) were far away from the human, indicating that there might be some intermediate host if they are a genuine original host.

### The Structure and Sequence Similarities Between the M2 Region of Angiotensin-Converting Enzyme 2 Protein in Humans and That in Other 30 Species Are Not Necessarily Consistent

We selected the amino acid sequences of the M2 region within ACE2 protein across 30 species (Supplementary Data Sheet 4) to predict their protein structures using the I-TASSER pipeline. We calculated the sequence and structural similarity of human ACE2 protein with that of the 30 species (Table 1). We performed a Spearman correlation test between the two vectors to test the consistency between sequence and structural similarity. The sequence and structure similarity metrics (including TM score, GDT-TS score, and RMSD) have a correlation of 0.379, 0.406, and −0.407 with a *p*-value of 0.039, 0.026, and 0.026, respectively, indicating that the two vectors are correlated thus statistically consistent. The top three species with similar structures to human ACE2 were *Vulpes vulpes* (TM score: 0.9831), *Pan paniscus* (TM score: 0.983), and *Ursus arctos horribilis* (TM score: 0.9828). However, the top three species ranked by sequence similarity are *Gorilla gorilla gorilla* (99.48%), *Pan paniscus* (99.48%), and *Pan troglodytes* (99.48%). Interestingly, *Rhinolophus sinicus* only has a sequence identity of 80.28% with the human in the M2 region of ACE2 protein, which ranks the last in the 30 species. However, its TM score is 0.9529, ranking 15th. These results indicated that variations in more amino acids in the protein sequence do not necessarily lead to more remarkable changes in structure.

**TABLE 1:** The sequence and structure similarities between the M2 region of ACE2 protein in humans and other 30 species.

Species	Sequence similarity (%)	TM score	GDT-TS score	RMSD
*Equus caballus*	87.2	0.9638	0.8434	1.705
*Gorilla gorilla gorilla*	99.48	0.9433	0.817	2.200
*Bos indicus x Bos taurus*	82.87	0.8199	0.4736	4.161
*Pongo abelii*	98.96	0.9662	0.8656	1.645
*Mustela putorius furo*	83.39	0.8673	0.6939	3.794
*Pan paniscus*	99.48	0.983	0.9112	1.154
*Bubalus bubalis*	82.87	0.8192	0.4745	4.171
*Octodon degus*	87.82	0.9647	0.8492	1.683
*Capra hircus*	83.22	0.8195	0.4745	4.167
*Mus musculus*	84.43	0.8577	0.6805	3.980
*Sus scrofa*	83.22	0.9663	0.8635	1.641
*Heterocephalus glaber*	88.41	0.8642	0.6922	3.802
*Rhinolophus sinicus*	80.28	0.9569	0.8308	1.885
*Felis catus*	85.99	0.9602	0.8484	1.803
*Bos mutus*	82.87	0.8199	0.4736	4.161
*Manis javanica*	85.47	0.8646	0.6784	3.806
*Chinchilla lanigera*	87.89	0.9562	0.8304	1.858
*Ovis aries*	83.05	0.8203	0.4837	4.151
*Erinaceus europaeus*	82.35	0.957	0.8279	1.878
*Ursus arctos horribilis*	85.49	0.9828	0.9133	1.156
*Panthera tigris altaica*	86.33	0.9827	0.915	1.159
*Pan troglodytes*	99.48	0.9679	0.866	1.598
*Vulpes vulpes*	84.79	0.9831	0.9146	1.143
*Sus scrofa domesticus*	82.87	0.9665	0.8626	1.635
*Macaca mulatta*	97.06	0.8642	0.6884	3.892
*Bos taurus*	82.87	0.8199	0.4736	4.161
*Chlorocebus sabaeus*	96.89	0.9666	0.8622	1.633
*Canis lupus familiaris*	84.97	0.9826	0.9095	1.161
*Oryctolagus cuniculus*	87.37	0.9562	0.8283	1.898
*Mesocricetus auratus*	87.72	0.8667	0.6889	3.789

### SARS-CoV-2 Might Infect a Few Species in Close Contact With Humans

The HADDOCK score estimates the stability of the complex of ACE2 protein binding with viral spike protein. A complex with HADDOCK scores less than 0 is considered stable. We showed the HADDOCK scores of the ACE2 protein from 31 studied species docking with spike protein in SARS-CoV-2 (Wuhan) and Omicron in Table 2. *Equus caballus* ranked first in the list, followed by *Oryctolagus cuniculus* for SARS-CoV-2 (Wuhan). In contrast, *Panthera tigris altaica* ranked first, followed by *Mus musculus* for Omicron. Previous studies have suggested that SARS-CoV-2 could infect Homo sapiens, *Panthera tigris altaica*, *Mustela putorius furo*, *Felis catus* and *Rhinolophus sinicus* rank 20, 10, 15, 5, and 6, respectively in the list of Wuhan. Thus, we suspect that the top 20 species are potentially susceptible animals for SARS-CoV-2 Wuhan. The 20 species include wild animals, husbandry animals, and pets, indicating that the infection of SARS-CoV-2 is unbiased across these three categories of animals.

**TABLE 2:** The HADDOCK scores of complexes formed by viral spike protein and the ACE2 proteins of 31 species.

Species	Genus	HADDOCK score [SARS-CoV-2 (Wuhan)]	HADDOCK score (Omicron)	Lower
***Manis javanica***	***Manis***	−99.5 ± 5.9	−161.9 ± 4.2	−**62.4**
***Mus musculus***	***Mus***	−128.7 ± 1.2	−171.7 ± 2.5	−**43**
***Panthera tigris altaica***	***Panthera***	−142.9 ± 6.2	−181.5 ± 4.4	−**38.6**
***Ursus arctos horribilis***	***Ursus***	−121.2 ± 8.2	−152.6 ± 4.1	−**31.4**
***Mustela putorius furo***	***Mustela***	−139.7 ± 4.5	−167.5 ± 7.5	−**27.8**
***Mesocricetus auratus***	***Mesocricetus***	−142.0 ± 1.3	−169.1 ± 5.4	−**27.1**
***Homo sapiens***	***Homo***	−**130.6 ± 6.4**	−**157.5 ± 4.6**	−**26.9**
*Canis lupus familiaris*	*Canis*	−123.7 ± 2.1	−146.1 ± 4.4	−22.4
*Vulpes vulpes*	*Vulpes*	−123.9 ± 7.8	−143.1 ± 9.7	−19.2
*Rhinolophus sinicus*	*Rhinolophus*	−145.9 ± 1.2	−164.3 ± 2.6	−18.4
*Heterocephalus glaber*	*Heterocephalus*	−143.8 ± 1.0	−161.5 ± 5.8	−17.7
*Pan paniscus*	*Pan*	−120.5 ± 2.4	−137.2 ± 5.5	−16.7
*Ovis aries*	*Ovis*	−131.3 ± 8.1	−146.3 ± 6.0	−15
*Pan troglodytes*	*Pan*	−130.6 ± 3.2	−145.2 ± 3.2	−14.6
*Macaca mulatta*	*Macaca*	−141.5 ± 7.6	−151.1 ± 2.3	−9.6
*Erinaceus europaeus*	*Erinaceus*	−143.9 ± 4.6	−153.3 ± 4.2	−9.4
*Octodon degus*	*Octodon*	−122.8 ± 0.6	−132.0 ± 3.2	−9.2
*Chlorocebus sabaeus*	*Chlorocebus*	−133.8 ± 3.3	−141.3 ± 5.2	−7.5
*Bubalus bubalis*	*Bubalus*	−145.3 ± 7.4	−150.5 ± 6.3	−5.2
*Chinchilla lanigera*	*Chinchilla*	−119.0 ± 5.2	−122.9 ± 1.8	−3.9
*Gorilla gorilla gorilla*	*Gorilla*	−134.0 ± 1.7	−137.7 ± 2.6	−3.7
*Capra hircus*	*Capra*	−142.4 ± 5.9	−145.3 ± 5.0	−2.9
*Sus scrofa domesticus*	*Sus*	−129.2 ± 6.3	−131.9 ± 3.6	−2.7
*Equus caballus*	*Equus*	−162.0 ± 10.2	−163.9 ± 3.1	−1.9
*Sus scrofa*	*Sus*	−120.1 ± 4.5	−121.0 ± 8.3	−0.9
*Oryctolagus cuniculus*	*Oryctolagus*	−149.1 ± 9.8	−148.1 ± 4.3	1
*Bos indicus x Bos taurus*	*Bos*	−141.5 ± 4.0	−140.2 ± 2.3	1.3
*Pongo abelii*	*Pongo*	−134.3 ± 8.3	−131.4 ± 2.1	2.9
*Bos taurus*	*Bos*	−146.8 ± 7.9	−141.9 ± 3.0	4.9
*Bos mutus*	*Bos*	−146.8 ± 7.9	−140.1 ± 1.9	6.7
*Felis catus*	*Felis*	−146.7 ± 8.3	−132.1 ± 5.9	14.6

*Bold characters represent species with increased binding affinity for ACE2 with SARS-Cov-2 Omicron, and numbers in bold indicate altered binding affinity.*

There is no significant correlation between ACE2 sequence similarity and ACE2-RBD structural similarity. The similarity of M2(ACE2)-RBD(SARS-CoV-2) structure between humans and other species was evaluated using RMSD and TM score, and the result is shown in Table 3. Surprisedly, compared with M2(ACE2)-RBD(SARS-CoV-2 Wuhan) structural similarity between human beings with other 31 species, M2(ACE2)-RBD(Omicron) decreased for most of the 31species.

**TABLE 3:** Structural similarity of M2 (ACE2)-RBD in *Homo sapiens* with other 30 species for SARS-CoV-2 (Wuhan) and Omicron.

Species	RMSD [SARS-CoV-2 (Wuhan)]	TM-score [SARS-CoV-2 (Wuhan)]	RMSD (Omicron)	TM-score (Omicron)
*Panthera tigris altaica*	1.77	0.96811	2.88	0.77747
*Pan paniscus*	2.01	0.9619	3.56	0.78573
*Sus scrofa*	2.3	0.94818	4.62	0.8361
*Bubalus bubalis*	2.35	0.9469	3.49	0.74715
*Pan troglodytes*	2.36	0.94803	2.86	0.77351
*Rhinolophus sinicus*	2.42	0.94378	3.12	0.7634
*Capra hircus*	2.44	0.94266	3.2	0.74733
*Octodon degus*	2.47	0.94334	3.14	0.77595
*Ovis aries*	2.48	0.94106	4.06	0.76453
*Felis catus*	2.58	0.93933	3.32	0.76102
*Ursus arctos horribilis*	2.68	0.7763	2.38	0.77636
*Chlorocebus sabaeus*	2.68	0.76621	3.32	0.7727
*Equus caballus*	2.72	0.93067	2.97	0.76931
*Bos mutus*	2.75	0.93056	3.43	0.75086
*Bos taurus*	2.75	0.93056	2.43	**0.94379**
*Vulpes vulpes*	2.75	0.7803	2.64	0.78087
*Chinchilla lanigera*	2.79	0.92399	3.01	0.75549
*Pongo abelii*	2.87	0.76351	3.58	0.778
*Bos indicus x Bos taurus*	2.9	0.92392	3.43	0.75085
*Canis lupus familiaris*	3.53	0.80209	2.43	0.77098
*Oryctolagus cuniculus*	3.72	0.88034	3.31	0.76199
*Macaca mulatta*	3.79	0.88881	4.47	0.70504
*Mustela putorius furo*	4.11	0.86043	4.26	0.70353
*Sus scrofa domesticus*	4.2	0.85671	3.44	0.77847
*Gorilla gorilla gorilla*	4.22	0.85136	3.56	0.76189
*Mesocricetus auratus*	4.38	0.84155	4.39	0.7052
*Heterocephalus glaber*	4.4	0.70857	4.5	0.70395
*Erinaceus europaeus*	4.5	0.84489	3.14	0.76817
*Manis javanica*	4.56	0.4939	4.41	0.72051
*Mus musculus*	4.63	0.70104	4.49	0.69368

*The bold number indicates that Bos taurus M(ACE2)-RBD(Omicron) has the highest structural similarity to Homo sapiens.*

### Human Infected SARS-CoV-2 Omicron Variant Originated From Another Animal Host?

**FIGURE 3:**
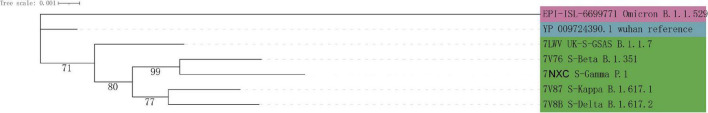
Phylogenetic tree of 7 SARS-CoV-2 variants based on their spike protein sequences. The branch length represents the number of substitutions per site, with the positions containing gaps and missing data being eliminated.

HADDOCK score of ACE2 protein docking with spike protein of Omicron decreased sharply for most 31 species studies. The top-eight species were *Manis javanica, Mus musculus, Panthera tigris altaica, Ursus arctos horribilis, Mustela putorius furo, Mesocricetus auratus, and Homo sapiens* (Table 2). In addition, a phylogenetic tree for 7 SARS-CoV-2 variants was constructed with spike protein sequences (Figure 3), showing an intriguing evolutionary relationship between Omicron with other variants that evolved in human patients. We speculated that the progenitor of Omicron rapidly evolved by accumulation of mutations conductive to infecting that host and then jumped into human beings.

Zhou et al. (2020) reported a cryo-electron microscopy structure of the complex, in which human ACE2 protein binds with the receptor-binding domain (RBD) of the viral spike protein. The structure indicated that human ACE2 protein interfaced with the RBD of viral spike protein by seven residues: Q24, D30, H34, Y41, Q42, K353, and R357 of ACE2 interact with Q474, K417, Y453, N501, Q498, N501, and T500 of RBD.

**FIGURE 4:**
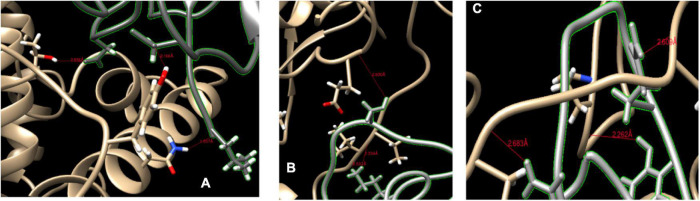
Binding sites of ACE2 protein and spike protein. **(A,B)** The binding sites of cat ACE2 and the SARS-CoV-2 RBD. **(C)** The binding sites of ferret ACE2 and the SARS-CoV-2 RBD. The red line represents the hydrogen bond.

At the N terminus of cat ACE2 protein (Figure 4A), we found that the ACE2 protein binds with RBD by three H-bonds. The THR739, TYR780, and ASN770 of cat ACE2 interacted with ASP428, ASP389, and LEU518 of RBD (Figure 4B). These H-bonds were less than 3 Å in the distance and provided a strong binding force.

The HADDOCK score of *Mustela putorius furo* was larger than that of *Felis catus*, and its ACE2 protein had six H-bonds with spike protein (Figure 4C). SER459 of RBD bond with GLU799 (2.8 Å) at the N terminus of the ACE2 of *Mustela putorius furo*. At the ACE2 loop, THR740 (2.334 Å), and ILE741 (2.532 Å) of ACE2 interfaced with LYS417 of the spike protein. LYS417, GLN474, and TYR489 of RBD loop bond with ILE741 (2.609 Å), ILE793 (2.626 Å), and PRO135 (2.638 Å) of ACE2.

### The Docking of the Receptor-Binding Domain of the Spike Protein to the Angiotensin-Converting Enzyme 2 Proteins Showed That the Binding Affinity Elevated in Subsequent Mutant Strains

**FIGURE 5:**
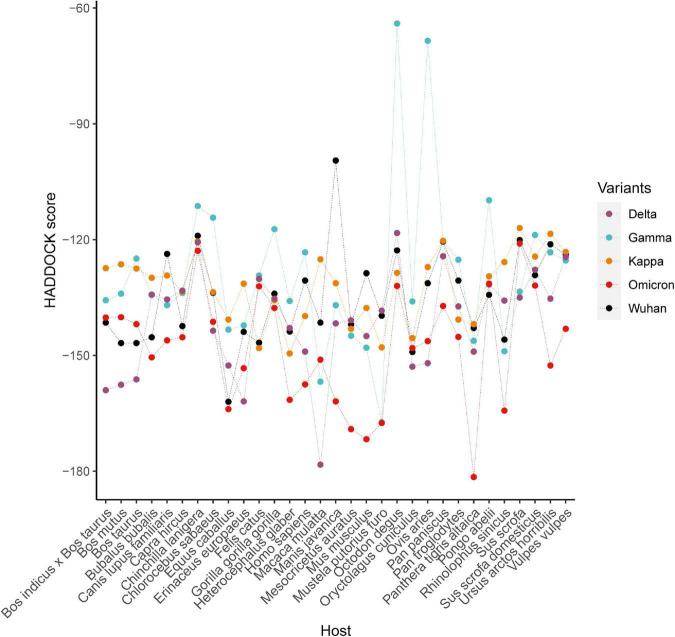
The HADDOCK scores of complexes formed by the ACE2 proteins of 31 species and the viral spike proteins from five SARS-CoV-2 strains.

Molecular docking between 31 ACE2 proteins and RBD of the Spike protein of SARS-CoV-2 were conducted for five strains (including Wuhan, Gamma, Delta, and Omicron), respectively. Of 31 species, 20 species exhibited the lowest value of HADDOCK score of ACE2 docking with Spike protein of Omicron and nine species for Delta variants (Figure 5), indicating that binding affinity of spike protein with ACE2 of host elevated with the accumulation of mutations in SARS-CoV-2 variants. For the Delta strain, the species with the strongest binding affinity to the Spike protein was *Macaca mulatta*. For the Omicron strain, the strongest binding affinity is *Panthera tigris altaica*.

The structural similarity of M2(ACE2)-RBD(SARS-CoV-2) between humans and other species were compared by the TMalign using metrics TM score and RMSD, and the results were shown in Supplementary Figures 1, 2, respectively. Surprisingly, compared with M2(ACE2)-RBD(SARS-CoV-2) structural similarity in the wild-type Wuhan strain, the similarity decreased in variants for most species of 31 animals (Supplementary Figures 1, 2). However, the most striking finding to emerge from our study was that structural similarity of M2(ACE2)-RBD(ARS-CoV-2) between *Homo sapients* and *Octodon degus* and was elevated for Delta virus compared with that of Wuhan strain. Furthermore, the structural similarity of M2(ACE2)-RBD(ARS-CoV-2) between Homo sapients and Bos taurus was elevated for the Omicron virus compared with that of the Wuhan strain.

### Human Angiotensin-Converting Enzyme 2 Protein Q340 Mutation Improves the Binding Affinity Between hACE2 and SARS-CoV-2

To test the effect of mutations of ACE2 on the binding affinity between hACE2 and SARS-CoV-2, we chose 11 residues to mutate and modeled the protein structure of the mutated ACE2 proteins. These residues were selected based on the frequency of mutations in other species (compared to humans). Table 4 shows the result of a few mutational ACE2 proteins docking with spike protein. The HADDOCK score of the original human ACE2 protein was −100.6 ± 17.5, which is less than the HADDOCK score of these mutated ACE2 proteins. This result indicates that the mutant ACE2 protein has a stronger binding capacity to the spike protein. Especially, the mutation Q340R has the lowest HADDOCK score of -139.2, which is the most stable in binding. It is known that residue 340 is not a binding site, suggesting that mutations in the non-binding sites can also affect the binding of ACE2 protein and spike protein. In the virus infection process from wild hosts to humans, SARS-CoV-2 may gradually adapt to the human ACE2 protein.

**TABLE 4:** The HADDOCK scores of mutated hACE2.

Position	Original residue	Mutated residue	HADDOCK score	±
40	F	S	−118.7	8
73	L	Y	−114.9	8.2
74	K	E	−115.1	7.2
78	T	K	−121.3	7.8
78	T	N	−106.7	12.9
78	T	R	−111.9	5.2
84	P	S	−112.3	2.7
95	L	R	−104.1	5.5
103	N	S	−106.8	11.1
110	E	A	−107.5	12.4
338	N	D	−116.8	13.6
339	V	G	−109.6	7.4
340	Q	R	−139.2	5.7

## Materials and Methods

### Blast Search on Species With Sequences Similar to Human Angiotensin-Converting Enzyme 2 Gene and the M2 Region of Human Angiotensin-Converting Enzyme 2 Protein

The amino acids (AA) sequence of the M2 region of hACE2 (1R42) was downloaded from the PDB database (PDBid: 1R42). The amino acid sequence was aligned with the NCBI nr database by blastp (Shiryev et al., 2007; Camacho et al., 2009). BLAST parameters were chosen as follows: e-value was set to 10; the cost of gap-open and gap-extend was set to 1; the maximum number of alignment results was set to 1E8. BLAST results with an e-value more than 0.01 and a similarity of less than 70% were filtered out.

### Selecting Candidate Species

Thirty-one species, including humans and 30 other species, were selected for further analysis according to four rules. Firstly, the similarity between the M2 region of human ACE2 protein and the given species was calculated; a species more similar than *Rhinolophus sinicus* was selected. We selected *Rhinolophus sinicus* as a borderline species since its infected coronavirus has 96% similhasy to SARS-CoV-2 at the whole-genome level, and it is not quite similar to humans. Secondly, six animals with phylogenetically identical to the human ACE2 gene were selected, including *Chlorocebus sabaeus*, *Gorilla gorilla gorilla*, *Macaca mulatta*, *Pan paniscus*, *Pan troglodytes*, and *Pongo abelii*. Thirdly, four animals, including *Felis catus*, *Mustela putorius furo*, *Panthera tigris altaica*, and *Manis javanica*, were selected since they are reported to be infected with SARS-CoV-2 or coronavirus with high similarity to SARS-CoV-2 (Shi et al., 2020). The selected 31 animal species cover wild animals, pet, and husbandry animals.

### Construction of Phylogenetic Tree

The ACE2 protein sequences of the 31 selected species were aligned by ClusterX (Thompson et al., 2002). Phylogenetic analysis was conducted using the maximum likelihood method based on the JTT matrix-based model (Jones et al., 1992). One thousand bootstrap replicates were used to calculate node support by MEGA7 (Kumar et al., 2016). The phylogenetic tree was displayed and annotated by the online tool iTOL (Letunic and Bork, 2019).

We constructed the phylogenetic tree for S proteins of 7 SARS-CoV-2 variants, including Wuhan, alpha, beta, gamma, kappa, delta, and Omicron, using the same approach as the ACE2 phylogenetic tree. Except for Omicron, the Spike protein sequences were retrieved from protein data bank (PDB) for six SARS-CoV-2 variants. The genome sequence for Omicron was downloaded from the GISAID database (Shu and McCauley, 2017), and the S protein sequence was annotated by local BLAST^2^.

### Modeling Angiotensin-Converting Enzyme 2 Protein Structure

The ACE2 protein structures of different species were modeled by the I-TASSR analysis, which consists of three steps, including (1) threading, (2) structural assembly, and (3) model selection and refinement (Roy et al., 2010; Yang et al., 2015). In the first step, LOMETS threaded the query protein sequence using a library of non-redundant structures to identify structural templates. In the second step, the topology of a full-length model was constructed by reassembling the continuously aligned fragments excised from templates. The structure of unaligned regions was built from scratch by *ab initio* folding. In the third step, the simulation of fragment assembly was utilized again as the starting selected cluster centroids. The final structural model was generated by building all-atom models from Cα traces using the optimized hydrogen-bonding networks. The similarity of human ACE2 protein structure and 30 species’ was calculated by TMscore program implemented in I-TASSER software using metrics TM score, GDT-TS score, and RMSD (Yang et al., 2015).

### Molecular Docking Between Angiotensin-Converting Enzyme 2 Protein and SARS-CoV-2 Spike Protein

The docking score of the HADDOCK server (van Zundert et al., 2016) was used to predict the binding affinity between SARS-CoV-2 spike protein and the ACE2 protein of a particular species. A few hACE2 residues, including Q24, D30, H34, Y41, Q42, K353, and R357, were set as the activated sites of the ACE2 receptor in HADDOCK since these residues were reported to effectively bind to the viral spike protein (Yan et al., 2020). In addition, the activated sites of spike protein included K417, Y453, Q474, Q498, T500, and N501, respectively.

Molecular docking between 31 ACE2 proteins and spike protein of five SARS-CoV-2 were conducted, respectively. The structural similarity of M2(ACE2)-RBD(SARS-CoV-2) between humans and other species were compared by the TMalign program implemented in I-TASSER software using metrics TM score and RMSD (Yang et al., 2015).

### Modeling the Mutation of hACE2 Protein

To predict the potential effect of mutants in hACE2, we mutate a few residues in hACE2, which have single nucleotide polymorphism (SNP) in 50% of species other than humans. Then, the Python library MODELER was used to model these mutated hACE2 proteins. Again, HADDOCK (van Zundert et al., 2016) was used to predict the binding affinity between the mutated/un-mutated hACE2 proteins.

## Discussion

The SARS-CoV-2 pandemic has already resulted in more than one hundred million human cases and caused a substantial economic loss. However, a detailed spectrum of susceptible animals to this virus is still unclear. In this study, we predicted susceptible animals to SARS-CoV-2 through a bioinformatics framework. Besides humans, we found that 22 species, including primate, pet, husbandry, and wild animals, were potentially susceptible to SARS-CoV-2, including cat, ferret, and tiger, which have already been reported to be naturally or experimentally infected with SARS-CoV-2.

We found some inconsistency among the amino acids sequence similarity of ACE2, the structural similarity of ACE2, and the binding affinity between ACE2 protein and SARS-CoV-2 spike protein across different species. The sequence similarity shows the evolutionary relationship among other species, while the structural similarity represents the similarity of protein folding (Zhang and Skolnick, 2005). Since the influence of each protein site is different, which might be possible that close evolutionary species exhibit quite different protein structures when important mutations occur (Zhang and Skolnick, 2005). Similarly, the inconsistency between structure similarity and binding affinity might be caused by the fact that the binding between ACE2 protein and viral spike protein only happens at active binding sites and the importance of each site is different. For example, *Canis lupus familiaris* (dog) has been reported to be infected by SARS-CoV-2 (Sit et al., 2020). The structural comparison showed that the ACE2 protein of dogs is quite different from that of humans. However, the docking results illustrated that ACE2 of dog can bind to the S protein of SARS-CoV-2. Therefore, we believe that protein-docking analysis is more reliable in predicting SARS-CoV-2 infection than sequence and structural similarity.

Though a few species, such as *Bos Taurus* have high docking scores, studies are suggesting that these species may not be infected by SARS-CoV-2 (Schlottau et al., 2020; Shi et al., 2020). Similar to SARS-CoV and MERS-CoV, the SARS-CoV-2 spike protein (S) has S1 and S2 (Li, 2016; Qing et al., 2020), with the S1 subunit containing the RBD region. Bloise et al. (2020) found that the infection of SARS-CoV-2 depends on both ACE2 and TMPRSS2 of host cells (Matsuyama et al., 2020). Specifically, after the S1 subunit of spike protein binds with ACE2 protein, the host TMPRSS2 protein cleavages the S protein into S1 and S2 subunits. The S2 subunit makes virus fusion with the host cell. We searched TMPRSS2 in the NCBI database and found that some species did not contain the TMPRSS2 gene, which might be why some species could not be infected even though their ACE2 has high docking scores with the S protein of SARS-CoV-2.

Our findings illustrated that the susceptibilities of canine, equine, and swine are a little bit higher than that of feline. However, these findings are not consistent with a previous study, which shows that feline belongs to the medium susceptible animal group, and swine, equine and canine fall into the low susceptible animal group (Damas et al., 2020). The possible reason for this discrepancy is that they ranked susceptible animals based on 25 known binding residues of ACE2 and its structure; however, we ranked susceptible animals according to HADDOCK score for the binding affinity between S protein and host ACE2. In the present study, we found that ACE2 of *Rhinolophus sinicus* showed low binding affinity to the S protein of SARS-CoV-2, which is consistent with Wu et al. (2020). In addition to Wu’s study, we also illustrated that *Heterocephalus glaber, Mesocricetus auratus, Chinchilla lanigera*, and *Ursus arctos horribilis* are susceptible animals to SARS-CoV-2. However, these prediction results should be experimentally confirmed in the future.

Biological methods screened susceptible animals through virus infection *in vivo* or pseudo-virus *in vitro*. However, they usually require labs with high biosafety levels and are time and cost-intensive. In addition, it is hard to catch wild animals and construct animal models (Bao et al., 2020; Bloise et al., 2020; Gand et al., 2020; Gorshkov et al., 2020; Hassan et al., 2020; Hou et al., 2020; Preziuso, 2020; Wang et al., 2020). Compared to computing methods, the advantage of biological methods is more accurate. In contrast, computing methods have advantages such as being fast, cheap, safe, and could predict wild animals. SARS-CoV-2 infection is a complex process (Hussain et al., 2020; Korber et al., 2020; Walls et al., 2020; Yan et al., 2020), which involves the interaction between viruses and hosts. Prevalent computing methods often consider one or two biological processing, such as binding and fusion, to predict the interaction between virus and host. Sometimes, a few results are inconsistent with biological experiments. For instance, *Erinaceus europaeus* was predicted to be more susceptible to SARS-CoV-2 than that of feline in our study; however, Wu’s research proved that pseudotyped SARS-CoV-2 fails to efficiently transduce into cells expressing ACE2 of European *hedgehog, lesser hedgehog tenrec* (Wu et al., 2020). Therefore, bioinformatics results need to be validated by biological assays.

## Conclusion

We illustrated that 23 animal species are potentially susceptible to the SARS-CoV-2 virus, including primates, companion pets, husbandry animals, and other wild animals, through a bioinformatics framework. These findings provide novel insight into tracing SARS-CoV-2, identifying susceptible animals, and controlling and preventing the SARS-CoV-2 pandemic.

## Data Availability Statement

The datasets presented in this study can be found in online repositories. The names of the repository/repositories and accession number(s) can be found in the article/Supplementary Material.

## Author Contributions

ML, AW, and JY designed the study. AW retrieved the data. AW and LW performed the data analysis. HS and AW wrote the manuscript. BW and GT reviewed the manuscript. All authors contributed to the article and approved the submitted version.

## Conflict of Interest

AW, LW, GT, and JY were employed by the company Geneis Beijing Co., Ltd. The remaining authors declare that the research was conducted in the absence of any commercial or financial relationships that could be construed as a potential conflict of interest.

## Publisher’s Note

All claims expressed in this article are solely those of the authors and do not necessarily represent those of their affiliated organizations, or those of the publisher, the editors and the reviewers. Any product that may be evaluated in this article, or claim that may be made by its manufacturer, is not guaranteed or endorsed by the publisher.

## Footnotes

1
https://coronavirus.jhu.edu/map.html

2
ftp://ftp.ncbi.nlm.nih.gov/blast/executables/blast+/LATEST

## Supplementary Material

Supplementary Figure 1 Structural similarity (TMalign Score) of M2 (ACE2)-RBD in *Homo sapiens* with other 30 species for five SARS-CoV-2 strains.

Supplementary Figure 2 Structural similarity (RMSD) of M2 (ACE2)-RBD in *Homo sapiens* with other 30 species for five SARS-CoV-2 strains.

Supplementary Data Sheet 1 Alignment results of human ACE2 protein sequence against the NCBI non-redundant protein sequences database.

Supplementary Data Sheet 2 ACE2 protein sequences of the 407 species from NCBI database.

Supplementary Data Sheet 3 ACE2 protein sequences of the 31 species from NCBI database.

Supplementary Data Sheet 4 Amino acid sequences of the M2 region within ACE2 proteins from 31 species.
