# Supplementary figures and images for: Systematic Tracing of Susceptible Animals to SARS-CoV-2 by a Bioinformatics Framework
Source: Front Microbiol. 2022 Mar 4;13:781770. doi: 10.3389/fmicb.2022.781770 (PMC8931700; doi:10.3389/fmicb.2022.781770)

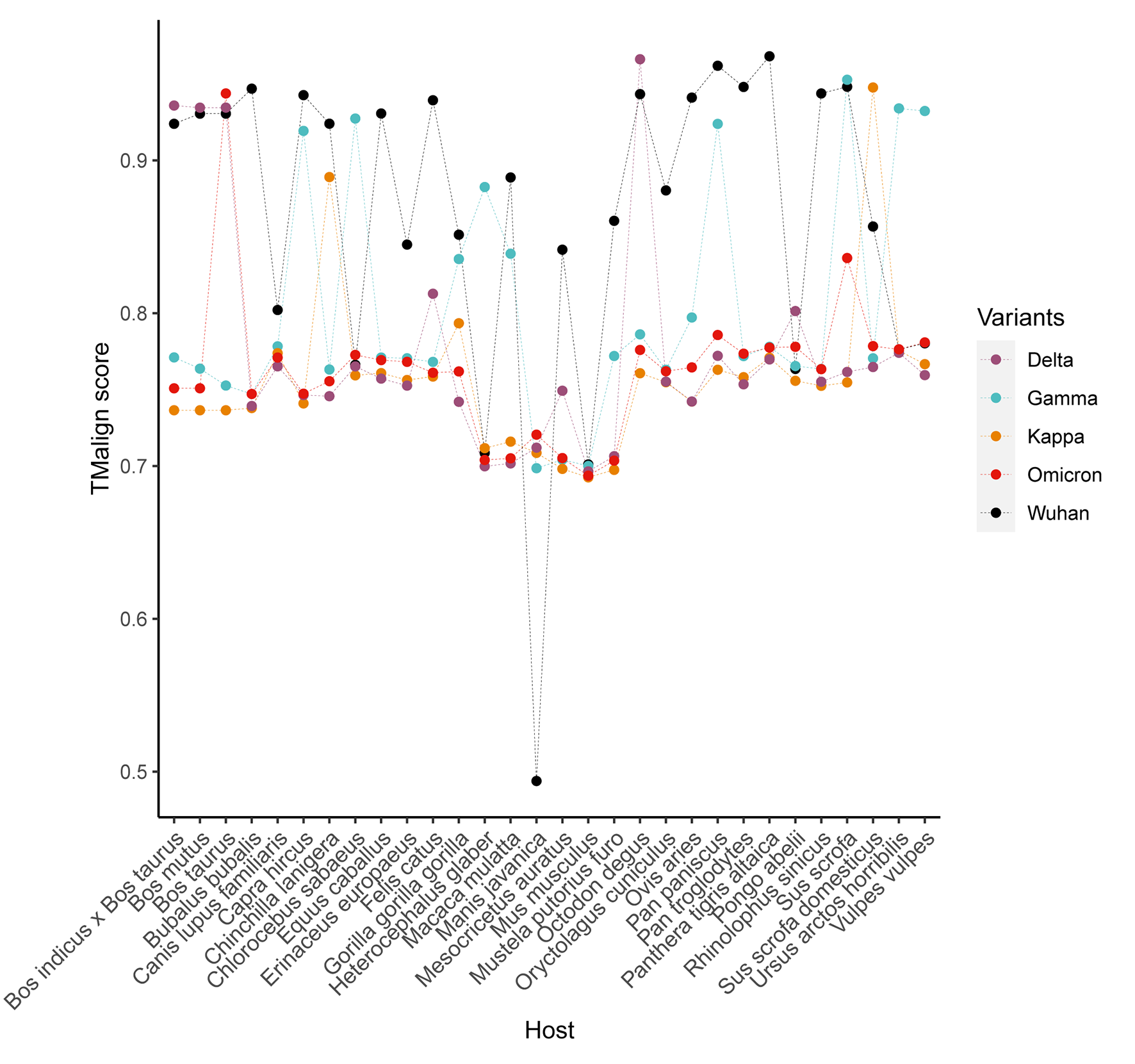

Supplement: Supplementary Figure 1 — Structural similarity (TMalign Score) of M2 (ACE2)-RBD in Homo sapiens with other 30 species for five SARS-CoV-2 strains. [file Image_1.TIF]

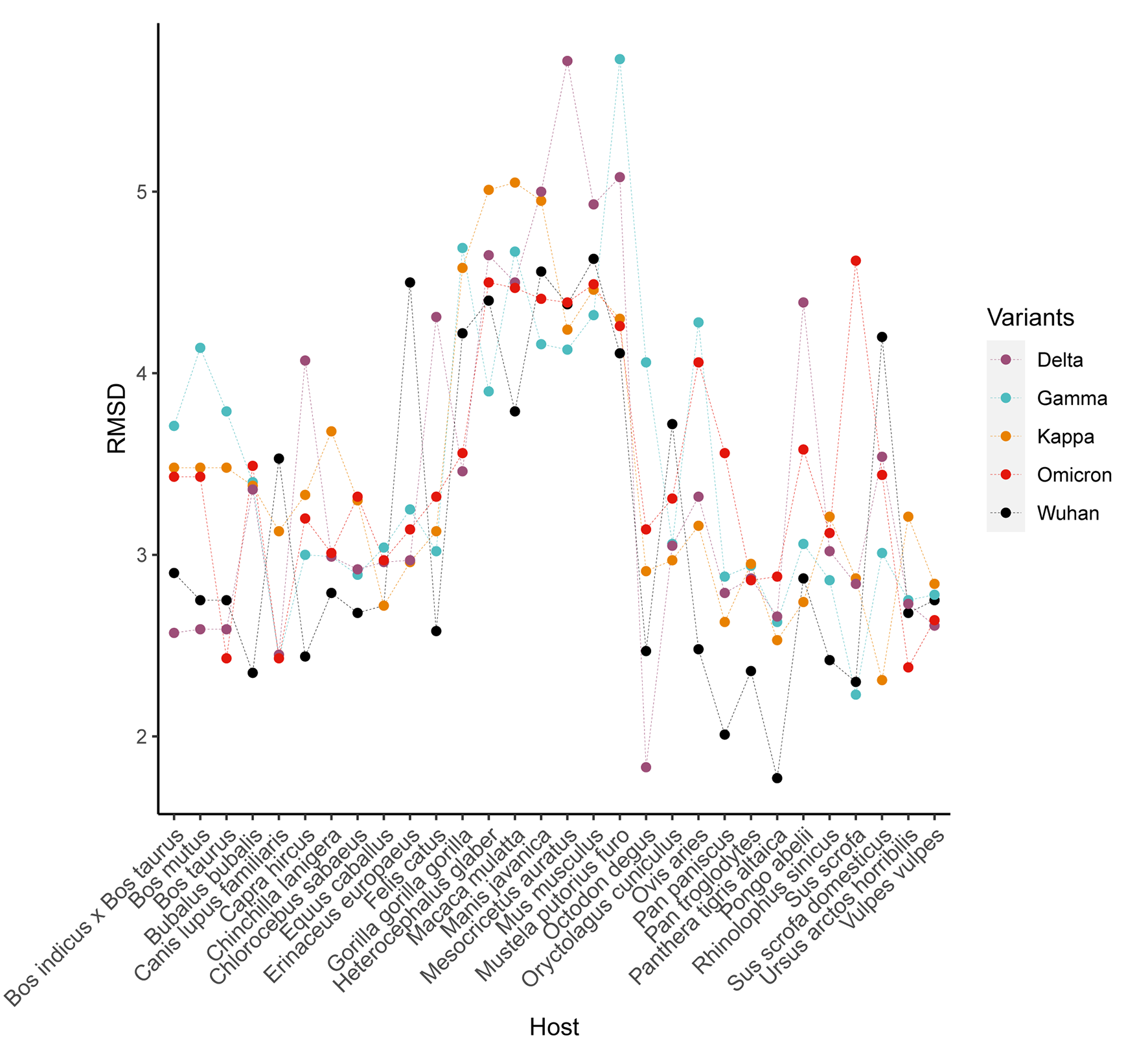

Supplement: Supplementary Figure 2 — Structural similarity (RMSD) of M2 (ACE2)-RBD in Homo sapiens with other 30 species for five SARS-CoV-2 strains. [file Image_2.TIF]
